# An fMRI investigation of the relations between Extraversion, internalizing psychopathology, and neural activation following reward receipt in the Human Connectome Project sample

**DOI:** 10.1017/pen.2020.11

**Published:** 2020-11-24

**Authors:** Courtland S. Hyatt, Emily S. Hallowell, Max M. Owens, Brandon M. Weiss, Lawrence H. Sweet, Joshua D. Miller

**Affiliations:** 1 Department of Psychology, University of Georgia, Athens, GA, USA; 2 Department of Psychiatry, University of Vermont, Burlington, VT, USA

**Keywords:** Extraversion, Reward processing, Five factor model, Internalizing psychopathology

## Abstract

Quantitative models of psychopathology (i.e., HiTOP) propose that personality and psychopathology are intertwined, such that the various processes that characterize personality traits may be useful in describing and predicting manifestations of psychopathology. In the current study, we used data from the Human Connectome Project (N = 1050) to investigate neural activation following receipt of a reward during an fMRI task as one shared mechanism that may be related to the personality trait Extraversion (specifically its sub-component Agentic Extraversion) and internalizing psychopathology. We also conducted exploratory analyses on the links between neural activation following reward receipt and the other Five-Factor Model personality traits, as well as separate analyses by gender. No significant relations (*p* < .005) were observed between any personality trait or index of psychopathology and neural activation following reward receipt, and most effect sizes were null to very small in nature (i.e., *r* < |.05|). We conclude by discussing the appropriate interpretation of these null findings, and provide suggestions for future research that spans psychological and neurobiological levels of analysis.

Extraversion is a broad, higher order personality trait domain that is associated with lower level facets such as gregariousness, dominance, and positive emotionality. Historically, Extraversion or constructs akin to it have been instantiated in many major models of personality (e.g., Eysenck & Eysenck, 1975; Tellegen, 1985; Wiggins, Trapnell, & Phillips, 1988), and there is strong, cross-cultural support for the positive relations between Extraversion and happiness, general positive affect, and subjective well-being (DeNeve & Cooper 1998; Diener, Sandvik, Pavot, & Fujita, 1992; Lucas, Diener, Grob, Suh, & Shao, 2000). One potential mechanism linking Extraversion to these adaptive outcomes is processing of reward (Smillie, 2013), which is consistent with physiological models of the agentic components of Extraversion that emphasize motivation for and sensitivity to environmental reward (Gray, 1970; Wilson, Barrett, & Gray, 1989). Multiple lines of empirical evidence support this Extraversion–reward link. For example, at the self-report level, Extraversion is related to overt reward-based constructs, such as sensitivity to reward (Mitchell et al., 2007) and reward responsiveness (Segarra, Poy, López, & Moltó, 2014). Extraversion is also related to a wide range of constructs adjacent to reward, including predicted levels of positive affect during a range of activities, as well as retrospective reports of enjoyment (Hoerger, Chapman, & Duberstein, 2016; Zelenski et al., 2013).

Beyond these domain-level links, there is evidence that sub-components of Extraversion are differentially related to these adaptive psychological outcomes that are thought to be largely rooted in experiences of reward (e.g., well-being). Specifically, enthusiasm (from the Big Five Aspects Scale; DeYoung, Quilty, & Peterson, 2007), which captures the communal components of Extraversion like positive emotionality and sociability, is more strongly related to most traditional indices of well-being such as life satisfaction, positive relationships, and feelings of meaning and mastery. Alternately, the facet assertiveness, which captures the agentic components of Extraversion like dominance, is more closely associated with feelings of autonomy (Sun, Kaufman, & Smillie, 2018). Overall, these lines of evidence suggest lower order aspects of Extraversion bear differential relations to these reward-adjacent constructs depending on how they are operationalized. In other words, these differential links may help clarify the nomological networks that support each level of the Extraversion trait hierarchy. Although the lower level trait components tend to “hang together” (i.e., be positively related to one another, bear similar relations to external criteria, etc.), it is important to examine them separately to determine the extent to which they converge and diverge in relations to important outcomes.

Another exemplary case of this complexity is the set of relations between Extraversion, its facets, and psychopathology, where the available literature indicates important relations at both the domain and facet level. At the domain level, Extraversion is concurrently, negatively related to a host of disorders of internalizing psychopathology, including dysthymic disorder, social phobia, panic disorder, major depressive disorder, post-traumatic stress disorder, and agoraphobia (Kotov, Gamez, Watson, & Schmidt, 2010).^1^ Moving away from traditional categorical diagnoses, quantitative models of psychopathology (i.e., HiTOP; Kotov et al., 2017) identify a construct akin to the low pole of Extraversion (called Detachment) as a major spectrum of psychopathology, and a construct deemed Detachment is also instantiated in the *DSM-5* Alternative Model of Personality Disorder diagnosis (American Psychiatric Association, 2013). Under this novel framework, trait elevations on Detachment can be used in support of a diagnosis of various personality disorders (e.g., avoidant personality disorder). Consistent with the links between Extraversion and well-being, the various processes (i.e., psychological, biological, social) that characterize Extraversion also appear to be protective factors against internalizing psychopathology. Psychotherapeutic intervention is associated with increases in Extraversion (Roberts et al., 2017), and in a recent randomized-controlled trial, participants assigned to “act-extraverted” condition (vs. control condition) experienced greater increases in positive affect (Jacques-Hamilton, Sun, & Smillie, 2019). Similarly, one of the most consistently supported psychotherapeutic interventions for depressive symptoms is behavioral activation, and a major element of behavioral activation treatments involves helping an individual increase the frequency with which they engage in pleasurable and rewarding events (Cuijpers, Van Straten, & Warmerdam, 2007; Lejuez, Hopko, Acierno, Daughters, & Pagoto, 2011), consistent with the notion that depression is due, in part, to a reduction of positive reinforcement (e.g., Lewinsohn, 1974; Lewinsohn & Graf, 1973).

More granular examinations have clarified that lower, more specific levels of the Extraversion hierarchy are better suited to capturing variance in aspects of internalizing psychopathology than the broader domain. In a recent review, Watson and colleagues (2019b) describe the differences that emerge in Extraversion – psychopathology relations as one moves “down” from the domain level to the facet level. A full review of these relations is beyond the scope of this manuscript, but several important trends emerged. First, both the agentic (e.g., dominance, assertiveness) and communal aspects of Extraversion (e.g., positive emotionality, enthusiasm) bore negative relations to self-report and interview ratings of depression, social dysfunction, as well as the pathological traits withdrawal, anhedonia, intimacy avoidance, and anxiousness measured by the Personality Inventory for DSM-5 (DeYoung, Carey, Krueger, & Ross, 2016; Krueger, Derringer, Markon, Watson, & Skodol, 2012). Notably, the communal components of Extraversion generally bore statistically significantly stronger correlations to these indices of internalizing psychopathology than the agentic components. Second, the agentic components of Extraversion bore medium to large, positive associations with certain pathological traits typically associated with externalizing psychopathology, including risk-taking, exhibitionism, manipulativeness, grandiosity, and attention-seeking. The communal components of Extraversion bore either small, positive, or null relations to these externalizing variables. In sum, multiple lines of evidence converge on the conclusion that Extraversion bears meaningful relations to various psychiatric disorders across methodologies (Watson et al., 2015), but these findings become more nuanced as one considers a more multi-faceted approach to measuring Extraversion and internalizing psychopathology.

## 1. The incentive facilitation model and extraversion-related reward

Given the well-established links between the facets of Extraversion and various indices of reward at the psychological level, a major initiative for personality researchers has been to investigate the degree to which these individual differences are linked to physiological responses to reward. Perhaps the most well-known neurobiological account of Extraversion (Depue & Collins, 1999) is specific to its agentic components (i.e., assertiveness, dominance); this model posits that the variation in the agentic components of Extraversion is rooted in variations in the incentive/reward motivational systems. Individuals high in agentic components of Extraversion are thought to be characterized by the tendency to encode the reward value of certain classes of stimuli more intensely, or to associate these stimuli with a larger incentive motivation. In other words, the agentic components of Extraversion are theorized to be related to encoding particular stimuli (e.g., interpersonal interactions) as relatively more rewarding, which then facilitates approach-related cognitions and behavior in an effort to attain this reward. Under this model, Depue and Collins (1999) argue that the agentic components of Extraversion – but not the more affiliative components like gregariousness and warmth – are underpinned by increased dopaminergic transmission in the mesolimbic dopamine pathway, which projects from the ventral tegmental area to regions such as the nucleus accumbens and orbitofrontal cortex. Put differently, this model postulates that agentic-extraverted individuals tend to exhibit higher levels of reward-related goal pursuit due, in part, to greater dopaminergic transmission in response to reward receipt. Thus, when agentic-extraverted individuals are placed in a context where they receive a reward, they may experience increased dopaminergic activation in the mesolimbic pathway because they have received a learning signal about the presence of reward in the environment, and/or they experience hedonic enjoyment (although there is evidence that opioid systems are more closely linked to hedonic enjoyment; Berridge, 2007).

Although this model was developed as a biological account of the agentic components of Extraversion, it has implications for other individual difference variables where reward is central to their conceptualization. Indices of internalizing psychopathology, especially depression, are thought to be related to diminished experience of reward (Whitton, Treadway, & Pizzagalli, 2015). For example, individuals with depressive symptoms appear to be less willing to exert effort to gain reward, and show reduced ability to integrate information about the probability of reward into their decision-making (Treadway, Bossaller, Shelton, & Zald, 2012). Perhaps most pertinently, anhedonia, or a pervasive, reduced ability to forecast or derive enjoyment from one’s experiences, is a central symptom in the *DSM-5* criteria for a diagnosis of Major Depressive Disorder (APA, 2013), and it is also a primary variable that distinguishes depression from other forms of internalizing psychopathology like anxiety (Clark & Watson, 1991). On the other hand, although anhedonia may be the depressive symptom that is most conceptually directly related to a lack of reward, recent evidence in youths suggests that anhedonia can be considered a transdiagnostic construct relevant to the expression of multiple depression and anxiety-related disorders (Conway, Li, & Starr, 2019). Although anhedonia is most traditionally considered a part of a latent depression variable, it appears pertinent to conceptualizing a range of indices of internalizing psychopathology.

### 1.1 Limitations of existing literature

Despite the comprehensiveness of this neurobiological model of the agentic components of Extraversion, to date, there have been few adequately powered examinations of the relations between Extraversion and reward-processing using functional Magnetic Resonance Imaging (fMRI) analysis (Allen & DeYoung, 2017; Yarkoni, 2015). Furthermore, though several existing investigations using electroencephalography speak to this relation, the findings are somewhat mixed. For example, in a longitudinal sample of children, self-report positive affectivity was related to feedback negativity (i.e., activation in the ventral striatum and medial prefrontal cortex in monetary loss trials minus activation in gain trials) in a hierarchical regression model controlling for demographic covariates and negative affectivity, but not at the bivariate level (*r* = −.09; N = 381; Kujawa et al., 2015). In adult samples, there is some recent evidence that reward-processing (i.e., activation in medial frontal regions during unpredicted reward trials minus unpredicted non-reward trials) is uniquely positively related to Extraversion (*r* = .26; N = 100; Smillie et al., 2019), but other studies have not found support for this relation (*r* = .06; N = 371; Suzuki, Hill, Ait Oumeziane, Foti, & Samuel, 2019).

In terms of functional neuroimaging methodology, there are numerous studies linking Extraversion to dopaminergic reward regions like the ventral striatum (Canli, Sivers, Whitfield, Gotlib, & Gabrieli, 2002; Canli et al., 2001; Cohen, Young, Baek, Kessler, & Ranganath, 2005; Mobbs, Hagan, Azim, Menon, & Reiss, 2005; Schaefer, Knuth, & Rumpel, 2011; Wu, Samanez-Larkin, Katovich, & Knutson, 2014), but virtually all of the previous work on Extraversion and fMRI reward-processing was statistically underpowered, with most studies using samples under 20 participants. This raises concerns regarding the stability (Schönbrodt & Perugini, 2013), replicability, and generalizability of these effects. A notable exception is a study by Civai and colleagues (2016), which examined the neural activation during selection between two payment options to assess the neural correlates of delayed reward decision-making (N = 250); Extraversion was not related to activation in any region assessed, which included reward-relevant regions such as the ventromedial prefrontal cortex and anterior cingulate cortex. However, it is possible that the decision-making aspects of making delayed reward discounting decisions may have modulated the activation of these regions in a different way than tasks involving simple reward receipt.

Investigations of the links between internalizing psychopathology (especially depression) and neural reward-processing have generally provided evidence that depression is related to reduced reward system activity during anticipation and receipt of reward (e.g., Hall, Milne, & MacQueen, 2014; Forbes et al., 2006; Forbes et al., 2010; Pizzagalli et al., 2009; Simon et al., 2010; Smoski et al., 2009). Unfortunately, much like the fMRI literature on Extraversion, these studies bear methodological limitations involving relatively small samples (i.e., all but one study under N = 100) and the use of extreme group designs (i.e., comparing individuals high and low in depressive symptoms). While this approach is intuitive, there are statistical ramifications to these designs that preclude estimation of the full population, including the portion of the population “in between” these groups (see Preacher, Rucker, MacCallum, & Nicewander, 2005). This is unfortunate because data suggest that depression is a dimensional rather than categorical construct (Hankin, Fraley, Lahey, & Waldman, 2005), and this measurement approach also limits the application of certain analyses (e.g., tests of non-linearity). In sum, while there is some precedent for relating internalizing psychopathology to alterations in reward-processing, well-powered analyses and dimensional measurement are needed.

### 1.2 Current study

Given these strong and consistent relations between Extraversion, its facets, and components of internalizing psychopathology, it has been posited that these constructs may be understood in terms of common neurobiological processes (DeYoung & Krueger, 2018), that is, complex phenotypes like Extraversion or internalizing psychopathology represent constellations of various psychological (i.e., affective, cognitive, motivational) processes that aggregate to represent a larger latent construct (e.g., DeYoung, 2015). Since each of these micro-features is, to some degree, tied to and substantiated by one’s physiology, a viable avenue for gaining a more robust understanding of the convergence/divergence of constructs is to investigate the neurobiological mechanisms that characterize them. Reward-processing is precisely this type of shared mechanism.

The goal of the current study was to examine the relations between Extraversion, two of its constituent facets (referred to as *Agentic Extraversion* and *Communal Extraversion*), a continuous measure of internalizing psychopathology, and neural activation following reward receipt using a functional neuroimaging methodology. We used existing data (N = 1050) from the Human Connectome Project (HCP) to investigate these relations (Van Essen et al., 2013a). To measure neural activation following reward receipt, we used a gambling task developed by Delgado and colleagues (2000, 2003). During this task, participants complete a card-guessing game wherein they receive feedback regarding the outcome of their guesses. In “reward” trials, feedback about correct guesses is accompanied by the receipt of $1.

To operationalize neural activation following reward receipt, we contrasted the activation in the brain’s reward system during reward trials to that during loss trials in which participants lose $.50, consistent with prior literature (Delgado et al., 2000; May et al., 2004; Tricomi, Delgado, & Fiez, 2004). Of note, we operationalized reward system regions in two ways. In the first approach, we used empirically defined regions of interest (ROIs) made from the activation of participants in the current study to reward outcomes. In the second approach, we used a priori defined ROIs identified using Neurosynth’s (https://neurosynth.org/) automated meta-analysis of studies operationalizing reward using task fMRI. The rationale for this dual approach was to examine our hypotheses in ROIs with high internal validity in the current sample (i.e., empirical ROIs), as well as ROIs consistent with prior literature in the interest of generalizability (i.e., a priori ROIs). By doing so, we aimed to minimize the chances that one ROI scheme was insufficient to detect any important effects (i.e., Type II error).

The primary hypothesis in the current study was that the domain Extraversion (as measured by the NEO-FFI; Costa & McCrae, 1992) would have a small (i.e., *r* = .10), positive relation to neural activation following reward receipt. We hypothesized that Agentic Extraversion would have a larger relation to neural activation following reward receipt than Communal Extraversion. Additionally, we hypothesized that internalizing psychopathology (as measured by the Achenbach Adult Self-Report scale of adaptive functioning; Achenbach & Rescorla, 2003) would have a small, negative relation to neural activation following reward receipt (as measured by the brain response to reward during the gambling task in reward-related ROIs; Delgado et al., 2000, 2003). A secondary hypothesis advanced was that the personality trait Neuroticism would also exhibit a small, negative relation to neural activation following reward receipt. This hypothesis was based on the preponderance of self-report-based evidence that Neuroticism is the personality trait most strongly linked to internalizing psychopathology (e.g., Kotov et al., 2010). In contrast to Extraversion and internalizing psychopathology, there have been remarkably few studies on Neuroticism and neural reward-processing (see reviews by Allen & DeYoung, 2017; Servaas et al., 2013), and, therefore, this secondary hypothesis was derived from these self-report findings rather than the fMRI literature. To test discriminant validity around these relations, we also conducted exploratory analyses on the relations between the other Five-Factor Model personality traits (i.e., Openness, Agreeableness, and Conscientiousness) and an index of externalizing psychopathology and neural activation following reward receipt. Like Neuroticism, there are very few studies on the relations between neural reward-processing and these other traits and externalizing psychopathology. Since we did not have *a priori* reasons to suspect relations based on prior literature, we expected null relations between neural reward-processing and Openness, Agreeableness, Conscientiousness, and externalizing psychopathology.

## 2. Methods

### 2.1 Participants

A sample of 1206 young adults was recruited as part of HCP (Van Essen et al., 2013a, 2013b). Informed consent was obtained for all participants. Participants were community-dwelling healthy adults between 22 and 35 years old with no significant history of psychiatric disorder, substance abuse, neurological disorder or damage, cardiovascular disease, or Mendelian genetic disease. Participants were included in the current analyses if they had valid data for all three of the major variable categories in the current investigation (i.e., personality, psychopathology, functional activation following reward receipt). Full fMRI reward data were initially available for 1081 subjects. However, we were notified on March 9, 2020, that an error has occurred with the processing of some of the fMRI data, which reduced this number to 1057 (https://wiki.humanconnectome.org/display/PublicData/HCP+Data+Release+Updates%3A+Known+Issues+and+Planned+fixes). An additional seven participants were missing personality and psychopathology data. This yielded a final sample of 1050 participants. Demographic information for this sample can be found in Table 1.

**Table 1.** Demographic information on the current sample (N = 1050)

**Sex**	
Male	45.7%
Female	54.3%
**Years of age**	28.8 (3.7)
**Race**	
White or Caucasian	75.0%
African American	14.5%
Asian American	5.8%
Native American	0.2%
More than one race	2.6%
Not sure or unknown	1.9%
**Ethnicity**	
Hispanic or Latino	8.7%
Not Hispanic or Latino	89.9%
Not sure or unknown	1.4%
**Years of education**	14.9 (1.8)

### 2.2 Materials and procedures

#### 2.2.1 Personality

Traits from the Five-Factor Model (FFM) of personality were measured using the NEO-FFI (Costa & McCrae, 1992), which is a 60-item measure that indexes characteristic patterns of thoughts, emotions, and behaviors. A reliability estimate was computed for each trait: Neuroticism (α = .84, ω = .84), Extraversion (α = .77, ω = .78), Openness (α = .75, ω = .76), Agreeableness (α = .76, ω = .77), and Conscientiousness (α = .81, ω = .83).

To test our hypotheses regarding Agentic and Communal Extraversion, we created composites using relevant items from the NEO-FFI. We compared the items from the NEO-FFI to the longer NEO-PI-R (Costa & McCrae, 1992) to identify which items on the NEO-FFI captured each of the Extraversion facets that are instantiated in longer measures (i.e., Warmth, Gregariousness, Assertiveness, Activity, Excitement-Seeking, Positive Emotions) assessed by this longer measure. To create the Agentic Extraversion variable, we included items that captured the Assertiveness (1 item) and Activity (3 items) facets of Extraversion, based on previous work (Gaughan, Miller, Pryor, & Lynam, 2009) describing the relations between the NEO facets and the agentic and communal aspects of Positive Emotionality as measured by the Multidimensional Personality Questionnaire (Tellegen & Waller, 2008). Notably, these results also suggested that the Achievement-Striving (3 items) and Self-Discipline (3 items) facets of Conscientiousness are also central to Agentic Extraversion, and indeed this is consistent with the original description of Agentic Extraversion provided by Depue and Collins (1999): “social dominance and the enjoyment of leadership roles, assertiveness, exhibitionism, and a subjective sense of potency in accomplishing goals” (p. 492). Thus, Agentic Extraversion was represented by 10 NEO-FFI items^2^ (α = .74, ω = .77). To create the eight-item Communal Extraversion variable (α = .73, ω = .75), we used NEO-FFI items measuring the Extraversion facets Warmth (1 item), Gregariousness (2 items), Excitement-Seeking (1 item), and Positive Emotions (4 items).

#### 2.2.2 Psychopathology

Psychopathology was measured using the Achenbach Adult Self-Report (ASR) scale of adaptive functioning, a 123-item measure of adaptive functioning for adults that includes items on emotional, behavioral, and social problems (Achenbach & Rescorla, 2003). Responses produce both internalizing and externalizing psychopathology composites which were used in the present analyses. The internalizing psychopathology composite comprises subscales for Anxiety/Depression, Withdrawnness, and Somatic Complaints, while the externalizing psychopathology composite includes Aggression, Rule-Breaking, and Intrusive Behavior subscales. Reliability estimates were computed for each index: Internalizing (α = .91, ω = .91) and Externalizing (α = .86, ω = .87).

#### 2.2.3 Reward-processing

The present study used an fMRI compatible adaptation of a gambling task developed by Delgado and colleagues to elicit neural activation following reward receipt (2000). In this task, participants are asked to guess whether the unknown number on a card is higher or lower than 5, and told that the accuracy of their guess that will result in a win or loss of money. Card numbers range from 1 to 9, and participants indicate their guess by pressing one of two buttons on the response box. Feedback about the number on the card is given as either 1) a green up arrow with “$1” for reward trials, 2) a red down arrow next to −$0.50 for loss trials; or 3) gray double-headed arrow for neutral trials (i.e., the number “5”). A fixed algorithm is used to ensure all participants experience the same number and pattern of wins and losses regardless of their guess (i.e., the task is “fixed”). In each of the two runs, there are two blocks of mostly reward (six reward trials pseudo-randomly interleaved with either one neutral and one loss trial, two neutral trials, or two loss trials) and two blocks of mostly loss (six loss trials interleaved with either one neutral and one reward trial, two neutral trials, or two reward trials), interleaved with four rest blocks in which participants passively view a fixation cross (15 s each). This results in a total of 32 reward events, 32 loss events, and four neutral events. All participants are paid the amount they won in the task (which is the same for all participants).

Participants have up to 1.5 s to respond, followed by feedback for 1 s. There is a 1-s interstimulus interval (ISI) in which a fixation cross is presented. Thus, one modeled event is 3.5 s total (response, feedback, ISI) with the only difference between reward trials and loss trials being the direction of the outcome. This modeling approach maximizes statistical power (i.e., samples multiple whole-brain images per trial), accounts for minor differences in hemodynamic response across participants by allowing for the capture of responses occurring slightly before or after the true “outcome” period which is only 1 s in duration, and is consistent with existing literature (Delgado et al., 2000; Forbes et al., 2006; May et al., 2004; Tricomi et al., 2004) and defaults published by the HCP. This task has been shown to reliably elicit activations in reward-related regions (Delgado et al., 2000; Forbes et al., 2006; May et al., 2004; Tricomi et al., 2004). Also consistent with prior work using this reward-processing task (Delgado et al., 2000; May et al., 2004; Tricomi et al., 2004), fMRI response during “win” trials (i.e., when $1.00 reward was received) was compared to response during “loss” trials (i.e., when participants lost $.50). As “neutral” trials (i.e., participants neither won nor lost) represented a very small proportion of trials (two neural trials per run, four total), they were not used as a baseline.

#### 2.2.4 Functional magnetic resonance imaging protocol

MRI was conducted using a 32-channel head coil on a 3 T Siemens Skyra (Siemens AG, Erlangen, Germany). T2* echoplanar fMRI data were collected during the gambling task (Delgado et al., 2000). A multi-band acceleration factor of 8 was used. Two task fMRI runs lasting 3:12 minutes each were completed with a TR = 720 ms, TE = 33.1 ms, flip angle = 52 degrees, FOV = 208 × 180 mm, and 72 2-mm-thick sagittal slices, resulting in 2.0-mm isotropic voxels (Barch et al., 2013). High-resolution T1-weighted structural images were acquired with a resolution of 0.7 mm^3^ isotropic (FOV = 224 × 240, matrix = 320 × 320, 256 sagittal slices; TR = 2400 ms and TE = 2.14 ms). The quality checking procedure completed to ensure all T1 scans were of high quality is documented in Marcus et al. (2013).

#### 2.2.5 Quantification of reward task fMRI response

Data were downloaded from the HCP database having been preprocessed using the minimal preprocessing pipeline (Glasser et al., 2013). This pipeline includes gradient unwarping, field-map-based EPI distortion correction, motion correction, registration of EPI to the structural scan and into MNI152 space, and grand-mean intensity normalization.

All subsequent fMRI data processing and analysis were then conducted using Analysis of Functional NeuroImages software (AFNI; Cox, 1996). Spatial smoothing was done to minimally preprocessed data using a 6-mm full-width half-maximum Gaussian filter. General linear modeling was conducted with regressors for the time course of events in each condition (win trials, loss trials, neutral trials), convolved with a hemodynamic response function, six dimensions of motion (x, y, z, roll, pitch, yaw), and linear, quadratic, and cubic trends. To capture reward-based hemodynamic response at the same point for all participants, the task was modeled as an event-related design with regressors individualized to each participant.

**Figure 1.**
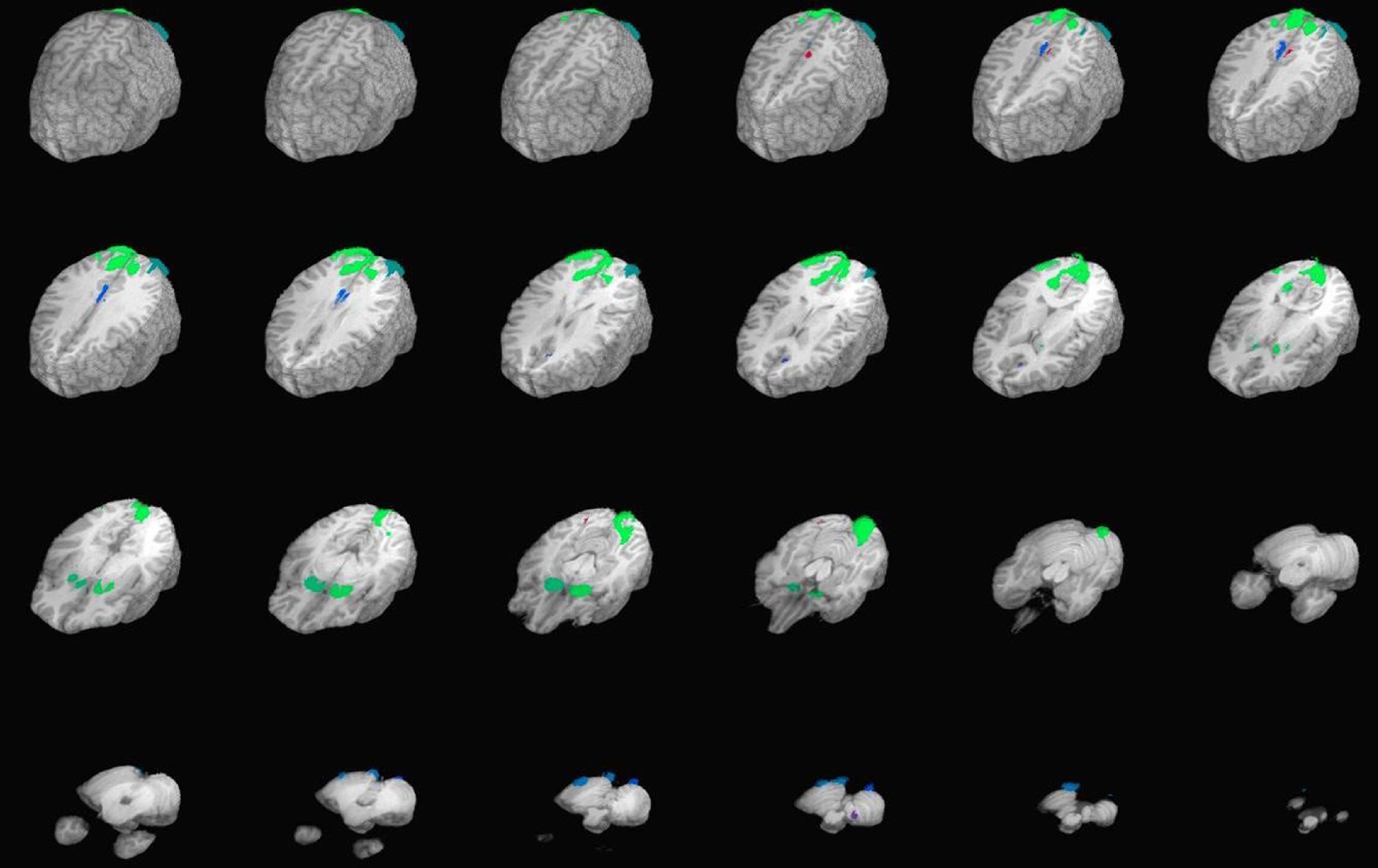
Empirically defined regions of interest from group-level activation of wins vs. losses. *Note:* Regions of interest exhibited significant activity during the win conditions on the gambling task compared to losses; Talairach Z-plane coordinates = +60 to −5-mm slices; thresholded to uncorrected *p* = 1^−45^

**Figure 2.**
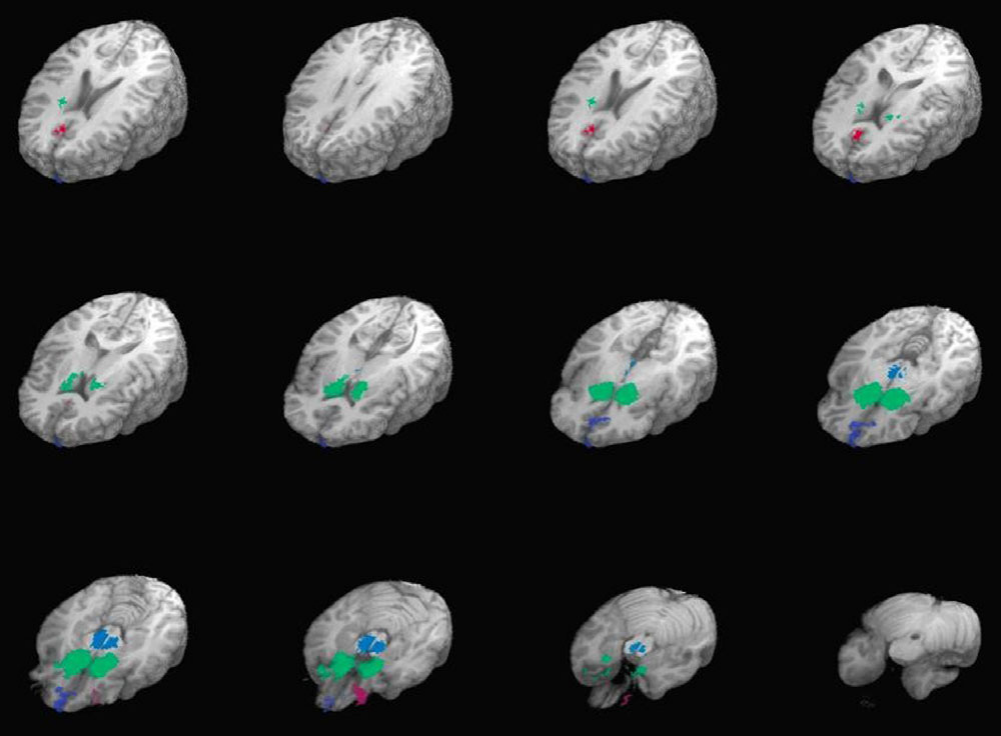
A priori defined regions of interest associated with reward-processing. *Note:* Brain regions associated with “reward” in Neurosynth Meta-analysis; Talairach Z-plane coordinates = +25 to −30 in 5-mm slices; thresholded to false discovery rate q = .05.

Empirically defined ROIs were created from group-level activation maps aggregating the contrast of win vs. loss trials across all participants (see Figure 1 and Table 2). Group summary activation maps for these ROIs were created using one-sample voxelwise t tests and thresholded to *p* < 1^−45^ with a cluster threshold of 20 voxels. This unusually stringent threshold was used to resolve separate core clusters of activation, as activation was robust throughout most of the brain. Average activation from all voxels of each ROI was extracted into SPSS (Version 24.0) for hypothesis testing. A priori defined ROIs were made using the term “reward” in Neurosynth’s automated lexical meta-analysis software found at https://neurosynth.org/ (see Figure 2 and Table 3). Average activation from all voxels of each ROI was also extracted for hypothesis testing into SPSS.

**Table 2.** Empirically defined regions of interest

Region of interest	# Voxels	Center of mass coordinates		
		*X*	*Y*	*Z*
R. cuneus	4915	−5.1	78.7	15.1
L. ventral striatum	628	15.6	−10.7	−5.1
R. ventral striatum	546	−15.8	−10.5	−5.8
L. middle occipital gyrus	459	29.1	81.5	25.1
R. cerebellum	381	−28.8	73	−43
R. posterior cingulate gyrus	265	−3.0	36.8	32.0
L. cerebellum	87	14.7	78.9	−42.7
L. anterior cingulate cortex	43	7.5	−40.0	14.5
L. cerebellar tonsil	42	18.4	42	−45.9
R. fusiform gryus	31	−30.5	75.9	−11.8
L. middle cingulate gryus	28	5.8	26.1	45.6
L. posterior cingulate gyrus	17	4.7	37.8	36.3

*Note.* L, left; R, right; center of mass coordinates are in Talairach space (RAI).

**Table 3.** A priori defined regions of interest

Region of interest	# Voxels	Center of mass coordinates		
		*X*	*Y*	*Z*
R. ventral striatum	1692	−15.2	−11.8	−5.8
L. ventral striatum	1415	12.7	−8.5	−5.4
R. thalamus/red nucleus	423	−6.2	18.1	−11.1
L. substantia nigra/red nucleus	258	6.2	16.7	−12.1
R. medial orbitofrontal gyrus	207	−5.0	−50.8	−8.2
L. middle frontal gyrus	151	19.0	−35.0	−15.4
R. anterior cingulate cortex	84	−5.0	−33.3	16.7
L. anterior cingulate cortex	73	6.2	−42/4	−4.8
L. medial orbitofrontal gyrus	31	2.3	−56.4	−8.0

*Note.* L, left; R, right; center of mass coordinates are in Talairach space (RAI).

### 2.3 Analyses

#### 2.3.1 Hypothesis testing

After establishing task-related ROIs, we computed Pearson’s correlations to examine the relations between Five-Factor Model personality traits, indices of psychopathology, and neural activation following reward receipt in this sample. As rates of psychopathology tend to differ for males and females, with females reporting higher rates of internalizing symptoms and males higher externalizing symptoms (Nolen-Hoeksema, 2012), we examined gender’s influence on relations of interest. Rather than using gender as a moderator in our analyses, we elected to repeat the above analyses with male and female subsamples.

#### 2.3.2 Power analysis

A power analysis was conducted using the pwr package in R (Champely, 2018). With N = 1050, a significant value = .005 for a two-tailed test, power estimates ranged from 67.0% (*r* = .10) to >99.9% (*r* = .20), suggesting the primary analyses were relatively well powered to detect small-to-medium effect sizes. The separate gender analyses were relatively underpowered to locate small effect sizes (*r* = .10) at this significance threshold (α = .005): 26.9% for the male subsample (N = 480) and 33.8% for the female subsample (N = 570). However, the separate gender analyses were relatively well powered for medium effects (*r* = .20) at this significance threshold (α = .005): 94.8% in the male subsample and 97.8% in the female subsample.

#### 2.3.3 Pre-registration note

Analyses were pre-registered prior to being conducted at https://osf.io/ysqmz. The data used in the current analyses had been processed as a part of the HCP minimal pre-processing pipeline prior to pre-registration and were downloaded and used by our group for studies unrelated to the topic of the current manuscript (i.e., reward-processing associations with ADHD symptoms, Owens et al., 2019b; structural correlates of personality and internalizing symptoms, Hyatt et al., 2019; Owens et al., 2019a). Correlations between the personality and psychopathology domains have been presented in manuscripts that predated the current project (e.g., Hyatt et al., 2019; Owens et al., 2019a). Group activation maps for the reward task used in the current manuscript are presented by Owens and colleagues (2019b). Consistent with recent recommendations (Benjamin et al., 2018), we pre-registered a threshold of *p* < .005 to determine the statistical significance of the relations between neural reward-processing and self-report variables (i.e., personality traits, psychopathology indices).

Three deviations from pre-registration were made in the present study. First, we pre-registered that we would conduct our analyses on N = 1075 since we had valid reward-processing data for N = 1081 but were missing valid personality and psychopathology data for N = 6. However, we discovered after pre-registration that we were missing valid personality and psychopathology data for N = 7, and that functional data for 25 participants were improperly processed; therefore, our final sample was slightly smaller than was pre-registered (N = 1050). Second, we did not pre-register that we would conduct analyses on Agentic or Communal Extraversion, but rather these were added based on a recommendation provided during peer-review.

The third deviation from pre-registration was that we pre-registered that we would conduct a series of multiple regression analyses, in which either the five FFM traits or the two psychopathology indices were entered simultaneously as predictors of the empirically or a priori defined ROIs. We planned to conduct these analyses to examine the relative predictive utility of the hypothesized predictors (i.e., Extraversion, Neuroticism, internalizing psychopathology) compared to the non-hypothesized predictors. However, given the consistently null findings observed in the current analyses, we chose to exclude these analyses, given that the predictive utility of all psychological-level variables individually was minimal. Another consideration that leads to this exclusion was Type I error: the large number of models that would be estimated for these multiple regression analyses (i.e., two sets of predictors [personality traits, psychopathology indices] X 21 ROIs [12 empirically defined ROIs, nine a priori defined ROIs] X three samples [full, men-only, women-only] = 126 additional models) would also considerably increase Type I error risk in the form of spurious suppressor effects while providing little potential benefit given the lack of effects found in individual predictor analyses.

## 3. Results

### 3.1 Relations between personality and psychopathology

Pearson’s correlation values for the relations between personality traits and psychopathology indices can be found in Table 4, and effect sizes are discussed such that small: *r* = |.10|, medium: *r* = |.20|, large: *r* = |.30|, and very large: *r* ≥ |.40| (Funder & Ozer, 2019). As expected, Neuroticism and Extraversion bore very large relations to internalizing psychopathology that were positive and negative, respectively, and Agreeableness and Conscientiousness bore very large and large (respectively), negative relations to externalizing psychopathology. The FFM personality traits displayed interrelations that were consistent with previous meta-analytic estimates (Van der Linden, te Nijenhuis, & Bakker, 2010). The internalizing and externalizing psychopathology composites displayed a very large, positive relation as expected (e.g., Kotov et al., 2017).

**Table 4.** Relations between personality traits and psychopathology indices

	1.	2.	3.	4.	5.	6.	7.	8.
1. Neuroticism	–							
2. Extraversion	−.34*	–						
3. Agentic Extraversion	−.39*	.59*	–					
4. Communal Extraversion	−.30*	.93*	.38*	–				
5. Openness	.02	.09	−.10	.12*	–			
6. Agreeableness	−.29*	.28*	.15*	.34*	.09	–		
7. Conscientiousness	−.39*	.27*	.79*	.17*	−.15*	.23*	–	
8. Internalizing	.67*	−.37*	−.36*	−.32*	.09	−.28*	−.30*	–
9. Externalizing	.39*	−.01	−.17*	−.01	.12*	−.46*	−.31*	.57*

*Note:* Internalizing and externalizing represent psychopathology composites of relevant subscales of the ASR; *p <.001.

### 3.2 ROI analyses relating reward-processing to personality and psychopathology

#### 3.2.1 Empirically defined ROIs

Pearson’s correlation values for the relations between reward-processing in empirically defined ROIs and personality traits and indices of psychopathology can be found in Table 5. In the full sample, no significant correlations were found between neural activity when receiving a reward and personality traits at the threshold *p* < .005, and the vast majority of effect sizes were null to very small in magnitude. Similarly, neither internalizing nor externalizing psychopathology was significantly correlated with neural activity when receiving a reward in the full sample.

**Table 5.** Pearson’s Correlations between fMRI reward task processing in empirically defined ROIs, personality, and psychopathology

	R. cuneus	L. ventral striatum	R. ventral striatum	L. middle occipital gyrus	R. cerebellum	R. posterior cingulate gyrus	L. cerebellum	L. anterior cingulate cortex	L. cerebellar tonsil	R. fusiform gyrus	L. middle cingulate gyrus	L. posterior cingulate gyrus
**N**	−.02	−.03	−.02	−.04	−.03	−.03	−.03	−.01	.02	−.01	.02	−.03
Male	−.03	.01	−.01	−.02	−.02	−.01	−.04	.02	.03	−.01	.03	.01
Female	−.02	−.07	−.02	−.05	−.04	−.05	−.02	−.02	.00	−.02	.03	−.07
**E**	−.04	−.02	−.02	−.02	.00	.03	−.05	−.02	−.02	−.02	.00	.03
Male	−.03	.00	−.02	.00	.01	.03	−.01	−.03	−.01	−.04	.03	.03
Female	−.05	−.04	−.02	−.04	−.01	.02	−.08	.00	−.02	−.01	−.04	.03
**Agentic E**	−.02	.03	.02	−.01	−.01	.01	.00	.00	−.01	−.02	−.04	.04
Male	.01	.07	−.01	.00	.02	.00	.04	−.01	.01	−.01	−.03	.06
Female	−.05	−.01	.05	−.02	−.04	.02	−.03	.00	−.02	−.03	−.04	.02
**Comm. E**	−.02	−.02	−.01	.00	.01	.01	−.05	.00	−.02	−.01	.02	.01
Male	−.03	.00	.00	.01	.01	.03	−.02	−.02	−.03	−.04	.05	.01
Female	−.02	−.03	−.03	−.02	.01	.00	−.08	.02	−.02	.01	−.01	.02
**O**	.04	.01	.00	.04	.07	.03	.02	.00	.03	.03	.05	.01
Male	.03	−.03	.02	.04	.06	.03	.02	.01	.00	.03	.03	.00
Female	.06	.05	−.02	.03	.09	.02	.02	−.02	.05	.04	.07	.02
**A**	.06	.00	.00	.05	.03	.01	.00	−.05	−.03	.06	.00	.03
Male	.12	.02	.06	.11	.09	.06	.08	−.02	.00	.09	.01	.06
Female	−.01	.00	−.05	.01	−.03	−.03	−.08	−.07	−.06	.03	.01	−.02
**C**	.02	.06	.04	.01	.00	−.01	.03	.01	.00	.01	−.03	.03
Male	.05	.09	.02	.01	.04	.00	.07	.00	.01	.03	−.02	.06
Female	−.02	.03	.07	.01	−.04	−.02	.00	.03	.00	−.01	−.04	.00
**Int.**	.01	−.01	.01	−.01	.01	−.01	−.02	.02	.06	.01	.03	−.03
Male	−.05	−.01	.02	−.05	−.05	−.03	−.11	.05	.06	−.01	.00	−.04
Female	.06	−.01	.00	.04	.07	.01	.05	−.01	.07	.03	.05	−.03
**Ext.**	−.05	−.03	−.03	−.02	.01	.00	−.05	.01	.02	−.04	.01	.00
Male	−.11	−.03	−.06	−.05	−.04	−.04	−.12	.03	.00	−.07	.02	.00
Female	.03	−.03	.01	−.01	.07	.04	.02	−.01	.04	−.01	−.01	.02

*Note:* L, left; R, right; values on the same row as the name of the personality trait or psychopathology index represent correlations presented for the entire sample (N = 1050); Male, correlations in the male subsample only (N = 480); Female, correlations in the female subsample only (N = 570); N, Neuroticism; E,Extraversion; O, Openness; A, Agreeableness; C, Conscientiousness; Int., internalizing psychopathology composite; Ext., = externalizing psychopathology composite.

#### 3.2.2 A priori defined ROIs

Pearson’s correlation values for the relations between reward-processing in a priori defined ROIs and personality traits and indices of psychopathology can be found in Table 6. Again, no significant correlations between reward-processing and personality traits or psychopathology indices were found at *p* < .005, and all effect sizes were null to very small.

**Table 6.** Pearson’s correlations between fMRI reward task processing in a priori defined ROIs, personality, and psychopathology

	R. ventral striatum	L. ventral striatum	R. thalamus/red nucleus	L. substantia nigra/red nucleus	R. medial orbito-frontal gyrus	L. middle frontal gyrus	R. anterior cingulate cortex	L. anterior cingulate cortex	L. medial-orbito-frontal gyrus
**N**	−.02	−.05	−.03	−.05	−.01	−.02	.00	−.01	−.03
Male	−.01	.01	−.05	−.11	.03	.00	−.01	.06	.02
Female	−.03	−.10	−.01	.00	−.06	−.03	.02	−.06	−.09
**E**	.00	−.02	.00	−.01	.02	.02	−.02	−.02	.01
Male	−.01	.00	.05	.02	.05	.02	.03	.00	.04
Female	.00	−.04	−.05	−.03	−.01	.03	−.06	−.03	−.01
**Agentic E**	.02	.03	.00	.01	.01	.02	−.04	.00	.03
Male	.01	.08	.06	.04	.04	.05	.04	.03	.08
Female	.02	−.01	−.05	−.02	−.03	−.01	−.10	−.03	−.01
**Comm. E**	.00	−.02	.01	.01	.03	.04	−.01	−.01	.01
Male	.00	−.01	.06	.04	.05	.02	.03	.01	.02
Female	.00	−.03	−.03	−.02	.01	.05	−.04	−.02	.01
**O**	.00	.01	.00	.05	.00	−.01	.00	−.01	.00
Male	.02	−.02	.02	.09	−.02	−.01	−.06	.00	−.01
Female	−.02	.03	−.02	.02	.03	−.01	.04	−.03	.01
**A**	.00	−.02	.02	.04	.00	.02	−.05	−.02	−.01
Male	.07	.00	.05	.13	.04	.03	−.04	.01	.05
Female	−.06	−.03	−.01	−.04	−.04	.00	−.07	−.04	−.06
**C**	.04	.06	−.01	.05	.00	.01	−.03	.01	.04
Male	.04	.10	.01	.09	.01	.02	.04	.04	.06
Female	.04	.03	−.03	.00	−.01	.00	−.08	.00	.02
**Int.**	.00	−.02	−.02	−.03	.00	−.02	.00	.01	−.01
Male	.01	−.01	−.05	−.11	.02	.00	.01	.05	−.01
Female	−.01	−.03	.00	.04	−.03	−.04	−.01	−.03	−.02
**Ext.**	−.03	−.02	−.01	−.02	.00	−.03	−.03	.01	−.01
Male	−.06	−.03	−.01	−.11	.00	−.01	−.04	.02	−.04
Female	.00	−.02	−.01	.07	−.01	−.05	−.03	−.02	.02

*Note:* Values on the same row as the name of the personality trait or psychopathology index represent correlations presented for the entire sample (N = 1050); Male, correlations in the male subsample only (N = 480); Female, correlations in the female subsample only (N = 570); N, Neuroticism; E, Extraversion; O, Openness; A, Agreeableness; C, Conscientiousness; Int., internalizing psychopathology composite; Ext., externalizing psychopathology composite.

## 4. Discussion

Despite clear links between Extraversion and self-reports of constructs that are thought to be related to reward (e.g., happiness, well-being, positive emotions, excitement-seeking; Costa & McCrae, 1980; Watson & Clark, 1997), empirical work on Extraversion and reward-processing from a neural level has been limited in previous investigations by small sample sizes and associated lack of statistical power (Button et al., 2013). In the current study, our primary aim was to investigate the hypothesized positive relations between Extraversion (specifically Agentic Extraversion) and neural activation following receipt of a reward, as well as the hypothesized negative relations between internalizing psychopathology and neural activation following reward receipt in large community sample (N = 1050). To address this question, we used two approaches to operationalizing the reward network ROIs (i.e., empirically defined and a priori defined) and conducted exploratory analyses in subsamples divided by gender (N_Men_ = 480, N_women_ = 570). In addition, the secondary aims of the current study were to test the hypothesized negative relations between neural reward-processing and Neuroticism, as well as to conduct exploratory analyses on the other FFM traits and externalizing psychopathology. We chose to use a threshold of *p* < .005 in this study as a way to balance Type I and Type II error risks (i.e., Benjamin et al., 2018), recognizing that this would enhance the risk of Type I error relative to more stringent methods of multiple-comparison correction (e.g., Bonferroni correction), which we deemed acceptable for testing associations in a network of regions with previously demonstrated association with Extraversion.

Contrary to hypotheses, we found no evidence for statistically significant (*p* < .005) relations between Extraversion and reward-processing in any of the empirically or a priori defined ROIs in the full sample, nor in the subsamples separated by gender. This same pattern of null findings was found for both Agentic Extraversion and Communal Extraversion variables. In terms of effect size, all of the relations observed between Extraversion, its facets, and reward-processing in an empirically or a priori defined ROI were null to very small in nature: across 189 examined effect sizes (three traits [Extraversion, Agentic Extraversion, Communal Extraversion] x 21 ROIs x three samples [full, men-only, women-only]), 183 (96.8%) were equal to or less than *r* = .05. Similar patterns were also observed for the relations between neural reward-processing and internalizing psychopathology, as well as neural reward-processing and Neuroticism: there were no statistically significant relations, and virtually all effect sizes were minuscule. Additionally, we conducted exploratory analyses on the relations between the other FFM traits and neural reward-processing, as well as on the relation between externalizing psychopathology and neural reward-processing. These non-hypothesized relations were also null.

This pattern of results is inconsistent with the predominant biological account of Agentic Extraversion (Depue & Collins, 1999) that hypothesizes that this trait is related to greater dopaminergic transmission following reward receipt. This presents a challenge to this model, and suggests that basic animal models of Agentic Extraversion and dopaminergic functioning may not be adequate for describing this phenomenon in humans. However, these results should not be overinterpreted to suggest that Extraversion and its facets are not related to experiences of reward – only that they appear unrelated to reward as operationalized in this narrow, fMRI task-based context. As previously detailed, there is a multitude of findings at the psychological level of analysis that suggests that self-reported experience of reward (and related constructs) are positively related to Extraversion and negatively related to internalizing psychopathology. Thus, we believe that psychological reward-processing is an empirically supported mechanism that links these constructs. However, the current results suggest that Extraversion, its facets, and internalizing psychopathology are not related to reward (relative to a baseline of a smaller loss) *at the neural level of analysis as measured by the fMRI task used herein.* This does not suggest that other elements of reward-processing in the brain (e.g., other neural networks) could not prove to be such a unifying mechanism.

### 4.1 Limitations

There are several key limitations that must be noted about the current investigation. First, the reward-processing task used provides a very specific operationalization of reward-processing: relative neural activation immediately following receipt of a reward (i.e., $1) in a given ROI compared to activation immediately following a smaller loss ($.50). This analytic strategy does not permit the identification of pure reward-related activation per se, but rather reward-processing compared to a baseline of a minor loss. The drawback to the current approach is that the fMRI signal used may be a consequence of “deactivations” due to losing as well as activations due to reward, which creates an ambiguity surrounding the interpretation of significant findings that cannot be disentangled from the data available. Although using reward trials compared to the neutral trials would represent a more focal way to isolate the intended effect, the limited number of neutral trials (i.e., 4 trials, <15 seconds) is not sufficient to reliably form a neural signal that can be used to compare to the reward trials. Additionally, it is possible that the relatively minor nature of the loss ($.50) did not render an acute loss response such that it significantly impacted the reward activation signal.

A second limitation involves psychometric concerns with the current reward-processing task. Recent work suggests that this task has relatively low test–retest reliability when examining ventral striatum activity in N = 45 participants from the HCP sample who were re-tested between approximately 2 and 7 months after the initial scan (Elliott et al., 2019). Although we examined different ROIs in a much larger sample, this type of critique is consistent with a broader current of field-wide criticism regarding the inadequate psychometric properties of many fMRI tasks (e.g., Elliott et al., 2019; Herting, Gautam, Chen, Mezher, & Vetter, 2018; Turner, Paul, Miller, & Barbey, 2018) as well as behavioral tasks and psychological measures more broadly (e.g., Enkavi et al., 2019; Hussey & Hughes, 2020). Thus, the current results must be understood with this important consideration in mind. Of note, this limitation is also present in essentially all prior research that did find associations between reward-related fMRI response and Extraversion or internalizing psychopathology, and thus represents a limitation of the field of fMRI as a whole rather than one specific to the current study.

Another important psychometric limitation pertains to the operationalization of Agentic Extraversion and Communal Extraversion. Although we believe we took a reasonable, empirically grounded approach to operationalizing these variables using the available NEO-FFI data, this measure was not developed to produce these subscales that we derived, and thus we acknowledge that more comprehensive assessments of Extraversion and its facets (e.g., Big Five Aspects Scale; NEO PI-R) would allow a more rigorous test of the hypothesized relations. An analogous limitation should be noted for the measure of internalizing psychopathology. We believe a more informative, rigorous test of the relations between internalizing psychopathology and neural activation following reward receipt would involve a more granular measure of psychopathology that permits examination of a broad internalizing domain alongside more specific symptoms (e.g., anhedonia, low mood). We considered creating these kinds of composites based on the item-level ASR but determined that there were not a sufficient number of relevant items to create sufficiently reliable and valid symptom composites, and thus our results cannot speak to these symptom-level relations.

A fourth limitation is that we are unable to parse the distinction between anticipatory and consummatory reward-processing, which is unfortunate since there are some theoretical reasons to suspect that Extraversion may be uniquely linked to anticipatory reward-processing (Smillie, 2013). Thus, despite the current null results, Extraversion may still be related to aspects of reward-processing in the brain, but limitations of the current reward paradigm do not allow the data to speak to this possibility. However, one strength of the study is that the ROIs used were based on two different operational definitions of the reward network, suggesting that idiosyncrasies of a single definition were not driving the lack of significant results. In a prior study (Owens et al., 2019a), the current task was shown to activate regions of the brain traditionally activated by reward outcomes in other task paradigms (Oldham et al., 2018). Although some of the empirically defined regions were not among those most commonly reported in the reward literature (e.g., bilateral cerebellum), key regions from the reward outcome literature (Oldham et al., 2018) were all represented, including the ventral striatum, ventromedial PFC/rostral ACC, and the posterior cingulate. Furthermore, the maps made from Neurosynth were based on a meta-analysis of 922 studies, suggesting they represent reward network regions broadly supported in the literature. Thus, although the task used in the present study cannot clearly differentiate between important elements of reward-processing, it does provide a relatively generalizable test of the relationship of Extraversion and internalizing psychopathology to the neural activation following reward receipt.

Another limitation is that although a priori power analyses suggested that statistical power to locate the hypothesized, small-to-medium effect sizes (*r* = |.10| to |.20|) was very high in the full sample (N = 1050), the power to locate small effects (*r* = |.10|) was quite low (i.e., below chance) for the subsample analyses separated by gender. At the time of pre-registration, we believed that a hypothesized effect size of *r* = |.20| for the current relations of interest was reasonable. In hindsight, this was a significant overestimation of likely effect sizes, which led us to conduct the gender subsample analyses when we were relatively underpowered to find true, small effects. We reported these gender subsample analyses to be consistent with our pre-registration and in the interest of transparency, but acknowledge that these analyses are not adequately powered to reliably locate the effects found here, and thus must be interpreted with caution. Finally, although our sample size was relatively large for this type of effort, participants were relatively physically and psychologically healthy (see exclusion criteria in Van Essen et al., 2013b). We believe it is essential to generalizability to examine these relations in samples characterized by higher rates of psychopathology, especially internalizing symptoms that are of most interest to this investigation.

### 4.2 Recommendations and future directions

As the corpus of evidence linking personality and psychopathology continues to grow, we believe that a thorough account of these constructs and the mechanisms that link them must involve multiple levels of analysis (e.g., biological, affective, cognitive, motivational, behavioral). However, the current research highlights some of the difficulties associated with this type of research: datasets large enough to conduct well-powered investigations across levels of analysis are scarce, the conclusions that one can draw from the fMRI tasks are often limited because they are designed to capture a very specific neural response, and the effect sizes of the relations between levels of analysis are likely to be very small (e.g., Poldrack et al., 2018). Given these obstacles, we are encouraged by the trend toward larger, open datasets (e.g., HCP, U.K. Biobank, Adolescent Brain Cognitive Development Study), which can be harnessed to address important questions about the neural underpinnings of complex phenotypes. Moreover, another potential pitfall is the lack of standardization of procedural components of task fMRI paradigms, such as stimulus design, timing, and instructions given to participants. We believe that efforts to better standardize fMRI tasks by making consensus best practice recommendations (e.g., Verbruggen et al., 2019 for the stop-signal task) is also a welcome development in addressing these issues; we are hopeful that similar initiatives will manifest for reward-processing tasks.

In terms of future directions, we believe that several of the limitations of the current work are illustrative of the types of issues that researchers are likely to encounter when pursuing this type of integrative project, and we hope these acknowledgements will contribute to the avoidance of similar issues in future data collection efforts. First, we emphasize the pressing need for a more thoroughly validated laboratory tasks that allow researchers to draw conclusions about important behaviors. This involves, at a bare minimum, establishing indices of construct validity in well-powered samples (e.g., Clark & Watson, 2019), and providing a detailed quantification protocol that permits an operational definition that is congruent with the conceptual definition of the construct that one is attempting to capture. Moreover, we reiterate the need for more precise measurement of psychological constructs as well. The personality and psychopathology literature provides strong support for hierarchical conceptualizations of these constructs, and, therefore, measures designed to capture them should reflect this complex structure. Specifically, personality measures that permit assessment of domains and facets, as well as psychopathology measures that capture both broader latent constructs and more granular symptoms, are strongly preferred.

A final, sobering perspective to consider is that the current results are consistent with the null or very small effect sizes reported in multiple separate efforts to link personality traits to structural and functional neural variables in large samples (e.g., Avinun, Israel, Knodt, & Hariri, 2019; Baranger et al., 2020; Gray, Owens, Hyatt, & Miller, 2018; Weiss et al., 2020). If one advocates a monist view of the brain and psychological functioning, these results can be difficult to square since this view implies that individual differences in emotions, motivations, behaviors, etc. necessarily emerge from a variety of physiological processes interacting with material elements of the environment. To compound this issue, this pattern of null findings does not appear to extend to all psychological individual differences, as there is recent evidence from a very large sample (N = 29,004) that intelligence bears a large relation (*r* = .28) to total brain volume, as well as other more specific regional correlates (e.g., insula volume; Cox, Ritchie, Fawns-Ritchie, Tucker-Drob, & Deary, 2019). So, what might account for the observed lack of relations for personality traits (beyond the limitations previously listed)? There are several additional considerations that may help reconcile these null findings with this existential position.

First, it is possible that personality traits (at the level currently assessed) are too broad, or encompass too diverse of an array of psychological processes to neatly and uniquely map onto a single neurobiological index. Given that personality traits capture wide swaths of thoughts, emotions, and behaviors over the lifespan, we believe this is a reasonable conclusion (also see Yarkoni, 2015), and we reiterate our previous point about the importance of measuring specific individual difference variables. Second, it is possible that the current neuroimaging tools are not sufficient to capture the critical inter-individual neural variation that is relevant to accounting for personality differences. Given the major advancements in neuroimaging technology in recent decades, equipment shortcomings seem to be a questionable and unsatisfying explanation. On the contrary, it may well be that researchers are just beginning to completely harness all of the sophisticated data gathered by modern neuroimaging devices. We believe that a potentially viable avenue for future research is to investigate the degree to which personality and psychopathology are related to neural variables of various operationalizations (e.g., single neuron; neural networks), which are becoming more accessible and analyzable.

Third, it is possible that age is a moderator of personality–neural activation relations, such that more substantial links emerge at earlier stages of development. Given that estimates of environmental influences on personality traits tend to increase across childhood and adolescence (e.g., Briley & Tucker-Drob, 2014), it is possible the neural responses to various environmental stimuli might demonstrate theoretically predicted relations in younger individuals who are still undergoing critical phases of social development and learning behavioral contingencies that exist in their interpersonal worlds. Fourth, we analyzed neural activation in response to reward receipt as a between-person variable based on a relatively simple series of stimuli (i.e., feedback about trial outcome). It is possible that a task paradigm with more intensive within-person assessment (e.g., assessing neural response to a range of stimuli of varying intensities) may yield a more robust and reliable operationalization of neural response to reward receipt. Of note, this appears less relevant for structural indices, which demonstrate excellent reliability (Elliott et al., 2019). Finally, a fifth possibility is that the effects of neural activity on personality are more readily identifiable “downstream” in neural circuitry. In other words, although the relation between a trait and relative degree of neural activation may be null when examined at the level of the brain, that neural activation may manifest in between-person variation in observable macro-level physiological (e.g., startle response, facial gestures) and cognitive (e.g., decision-making, selective attention) processes that are more directly and meaningfully tied to personality traits and/or indices of psychopathology.

### 4.3 Conclusions

In the largest sample to date, the personality domain Extraversion, its sub-components Agentic Extraversion and Communal Extraversion, as well as index of internalizing psychopathology were not significantly associated with neural activation following receipt of a reward. These results were inconsistent with theoretical explanations of the role of reward-processing in personality and psychopathology, but are consistent with other recent evidence that the relations between personality and the brain are, as currently measured, null or very small. We believe this work highlights the importance of clear operationalizations of complex constructs when investigating biological underpinnings, and we are hopeful that careful psychometric considerations will improve our search to better understand the biological substrates of personality and psychopathology.

## Acknowledgments and Conflicts of Interest Declaration

The data used in this project are from the Human Connectome Project, WU-Minn Consortium (Principal Investigators: David Van Essen and Kamil Ugurbil; 1U54MH091657) funded by the 16 NIH Institutes and Centers that support the NIH Blueprint for Neuroscience Research; and by the McDonnell Center for Systems Neuroscience at Washington University in St. Louis. The authors are deeply appreciative of the Human Connectome Project for open access to its data.

At the time of submission, the authors have no conflicts of interest to declare.

## Footnotes

1 To add to this complexity: while not reviewed extensively here, high levels of Extraversion have also been specifically linked to aspects of mania such as elation and excess energy (Watson et al., 2019a, 2019b). Moreover, Extraversion has been linked to personality disorder constructs typically associated with externalizing psychopathology, namely narcissism and psychopathy. Extraversion is related to the agentic, grandiose aspects of narcissism (Paulhus, 2002), which is seen as central to this construct by academicians, clinicians, and lay persons (Miller, Lynam, Hyatt, & Campbell, 2017). Additionally, historical writings (Cleckley, 1941) and contemporary models of psychopathy (e.g., Triarchic Model of Psychopathy; Patrick, Fowles, & Krueger, 2009) identify a construct called “Boldness” or “Fearless Dominance,” which can be viewed as an admixture of high Extraversion and low Neuroticism (e.g., Hyatt, Crowe, Lynam, & Miller, 2020).

2 The NEO-FFI items included in the Agentic Extraversion variable are items 10, 25, 30, 32, 35, 47, 50, 52, 57, and 60. The NEO-FFI items included in the Communal Extraversion variable are items 2, 7, 12, 17, 22, 27, 37, and 42.
